# Untargeted Metabolomics Sheds Light on the Secondary Metabolism of Fungi Triggered by Choline-Based Ionic Liquids

**DOI:** 10.3389/fmicb.2022.946286

**Published:** 2022-07-25

**Authors:** Patrícia Sequeira, Maika Rothkegel, Patrícia Domingos, Isabel Martins, Céline C. Leclercq, Jenny Renaut, Gustavo H. Goldman, Cristina Silva Pereira

**Affiliations:** ^1^Applied and Environmental Mycology Laboratory, Instituto de Tecnologia Química e Biológica António Xavier, Universidade Nova de Lisboa (ITQB-NOVA), Oeiras, Portugal; ^2^Integrative Biology Platform, Environmental Research and Technology Platform, Luxembourg Institute of Science and Technology, Belvaux, Luxembourg; ^3^Faculdade de Ciências Farmacêuticas de Ribeirão Preto, Universidade de São Paulo, Ribeirão Preto, Brazil

**Keywords:** *Neurospora crassa*, *Aspergillus nidulans*, *Aspergillus fumigatus*, non-proteinogenic amino acids, antimicrobial compounds, peptidome

## Abstract

Fungal secondary metabolites constitute a rich source of yet undiscovered bioactive compounds. Their production is often silent under standard laboratory conditions, but the production of some compounds can be triggered simply by altering the cultivation conditions. The usage of an organic salt – ionic liquid – as growth medium supplement can greatly impact the biosynthesis of secondary metabolites, leading to higher diversity of compounds accumulating extracellularly. This study examines if such supplements, specifically cholinium-based ionic liquids, can support the discovery of bioactive secondary metabolites across three model species: *Neurospora crassa, Aspergillus nidulans*, and *Aspergillus fumigatus*. Enriched organic extracts obtained from medium supernatant revealed high diversity in metabolites. The supplementation led apparently to increased levels of either 1-aminocyclopropane-1-carboxylate or α-aminoisobutyric acid. The extracts where bioactive against two major foodborne bacterial strains: *Staphylococcus aureus* and *Escherichia coli*. In particular, those retrieved from *N. crassa* cultures showed greater bactericidal potential compared to control extracts derived from non-supplemented cultures. An untargeted mass spectrometry analysis using the Global Natural Product Social Molecular Networking tool enabled to capture the chemical diversity driven by the ionic liquid stimuli. Diverse macrolides, among other compounds, were putatively associated with *A. fumigatus*; whereas an unexpected richness of cyclic (depsi)peptides with *N. crassa*. Further studies are required to understand if the identified peptides are the major players of the bioactivity of *N. crassa* extracts, and to decode their biosynthesis pathways as well.

## Introduction

Microbial infections and antimicrobial resistance constitute globally a major threat to human health. The last was recognized by the World Health Organization, in 2019, as one of the top 10 global public health threats facing humanity. It is estimated that *ca.* 700,000 people die every year from drug-resistant infections (World Health Organization [WHO], 2021). To fight this threat, the development of new drugs that target microbial virulence and/or pathogenicity is a priority (Meyer et al., 2016). Microorganisms constitute a diverse and resourceful source for bioactive natural products discovery, which can be used as drug leads or therapeutics itself (Newman and Cragg, 2020). In particular, filamentous fungi are considered gifted producers of structurally diverse low-molecular weight secondary metabolites. These compounds are synthesized by using precursors derived from primary metabolism and, generally, are not essential for the growth and development of the producer organism (Fox and Howlett, 2008; Brakhage, 2012; Netzker et al., 2015). Secondary metabolites are, however, often critical for the survival and growth of the fungus in its ecological niche (Fox and Howlett, 2008; Rodrigues, 2016), with roles identified for example in nutrient acquisition, interaction with other organisms and growth inhibition of competitors (Calvo et al., 2002; Khaldi et al., 2010; Brakhage, 2012; Macheleidt et al., 2016).

Fungal secondary metabolites classes comprise polyketides (PKs), non-ribosomal peptides (NRPs), PK-NRPs hybrids, indole alkaloids, and terpenes (Pusztahelyi et al., 2015; Bills and Gloer, 2016). PKs, the most abundant class, use acetyl-CoA and malonyl-CoA units, and biosynthesis is simply achieved by the elongation of carboxylic acid building blocks. The scaffold is further modified by oxygenases, glycosyltransferase and other transferases leading to a high degree of structural diversity (Hertweck, 2009; Brakhage, 2012). NRPs, the second largest class, are synthesized by the modular assembly of short carboxylic acids and/or amino acids (El Maddah et al., 2017). They are constituted of both proteinogenic and non-proteinogenic amino acids and show high diversity in terms of length, variation in their functional domains and whether they are cyclized or not (Keller et al., 2005). Other units such as fatty acids, α-hydroxy acids, α-keto acids, heterocycles, and others, can also be incorporated (McErlean et al., 2019). Terpenes(oids) are made up of several C5 isoprene units, which are synthesized from acetyl-CoA through the mevalonate pathway. They are found to be linear or cyclic, saturated or unsaturated. Their classification is based on the number of isoprene units, among others, triterpenes (steroids) and tetraterpenes (carotenoids) (Bhattarai et al., 2021). Compounds of pharmacological interest are for example griseofulvin – PKS (Cacho et al., 2013) and echinocandin B – NRP (Cacho et al., 2012), both with antibiotic properties, and fumagillin – terpenoid, with potential antifungal and antitumoral properties (Lin et al., 2013).

The first biosynthesis step is catalyzed by a multidomain (backbone) enzyme that defines the produced class: PKs synthases, NRP synthases, hybrid NRP–PK synthases, prenyltransferases (or dimethylallyl tryptophan synthases), or terpene cyclases (Keller, 2019). Genes encoding for biosynthesis of a secondary metabolite are often arranged in gene clusters that are co-regulated under certain conditions; usually silent under standard laboratory conditions (Brakhage, 2012). Many backbone genes already identified have not yet been matched to the produced compound, and *vice versa* (Bergmann et al., 2007; Brakhage, 2012). To stimulate the production of a rich diversity of secondary metabolites, several strategies have been used, for example co-cultivation with other fungi/bacteria or genome engineering (Netzker et al., 2015; Begani et al., 2018; Liu et al., 2021). As illustrative examples, temperature modulates the production of trypacidin and endocrocin in *A. fumigatus* germinating spores, whereas white light represses the production of aflatoxin and sterigmatocystin in *A. fumigatus* (Hagiwara et al., 2017) and of the later metabolite in *A. nidulans* (Bayram et al., 2008). The simplest is, however, the one strain-many compounds (OSMAC) approach that explores modification of the cultivation conditions to activate those metabolic pathways (Bode et al., 2002). Ionic liquids, organic salts with a melting point below 100°C, represent a promising class of chemical stimuli that can profoundly impact fungi metabolism (Petkovic et al., 2009; Martins et al., 2013; Alves et al., 2016; Hartmann et al., 2019). When used as growth media supplements, many backbone genes underwent upregulation and a higher diversity of secondary metabolites, including cryptic ones, were biosynthesized (Martins et al., 2013; Alves et al., 2016). The stimuli caused by the ionic liquid supplements differ from that of a simple inorganic salt (Petkovic et al., 2010). As an example, in *A. nidulans*, orselinic acid, which has been identified in ionic liquid supplemented cultures (Alves et al., 2016), is also produced during co-cultivation with *Streptomyces* spp. that modulates the epigenetic machinery of the fungus (Bayram et al., 2019).

This study examines if ionic liquids supplements can support discovery of bioactive secondary metabolites in fungi. Three model fungi – *Neurospora crassa, Aspergillus nidulans*, and *Aspergillus fumigatus*, and two choline-based ionic liquids – choline chloride (ChoCl) and choline decanoate (ChoDec), were tested. Specifically, we focused on compounds accumulating extracellularly. The antibacterial activity of the ensuing crude extracts was evaluated against two major foodborne bacterial strains, *Staphylococcus aureus* and *Escherichia coli*. To characterize the chemical landscape of the extracts, their amino acid composition and an untargeted mass spectrometry analysis using the online platform Global Natural Product Social Molecular Networking – GNPS – were applied. An unexpected richness of peptide-based structures could be putatively associated with *N. crassa*.

## Materials and Methods

### Chemicals

Compounds used in preparation of minimal media were purchased from Sigma-Aldrich, except for NaCl and MgSO_4_⋅7H_2_O (Panreac), phosphoric acid (Fisher Scientific) and NaNO_3_ (ACROS organics). The standard chemicals [1-aminocyclopropane-1-carboxylate (ACC) and α-aminoisobutyric acid (Aib)] and chromatographic solvents were of highest analytical grade and purchased from Sigma Aldrich and Fisher Scientific, respectively. Water was obtained from a Milli-Q system (Millipore). Choline Chloride (>98%; ChoCl) was purchased from Sigma Aldrich and Choline Decanoate (>95%; ChoDec) from Iolitec.

### Fungal Strains

*Aspergillus fumigatus* AF293 (FGSC A1100), *A. nidulans* (FGSC A4) and *N. crassa* (FGSC 2489) were obtained from the Fungal Genetics Stock Center. All strains were cultivated on DG18 (Oxoid) agar plates. Cultures were incubated in the dark, for 6–7 days, at 30°C (*A. nidulans* and *N. crassa*) or 37°C (*A. fumigatus*). Asexual spores (conidia) were harvested using a NaCl (0.85% w/v) and Tween-20 (0.1% w/v) sterile solution and collected after passing through three layers of miracloth. The harvested spores were washed twice with a sterile NaCl solution (0.85% w/v) and finally resuspended in the NaCl solution (0.85% w/v), to be used immediately, or in a cryoprotective saline solution containing 10% (v/v) glycerol, to be stored at −20°C or −80°C.

### Growth Media

*Aspergillus fumigatus* and *A. nidulans* were cultivated in liquid minimal medium containing glucose (10.0 g⋅L^–1^), thiamine (0.01 g⋅L^–1^), 5% (v/v) nitrate salts solution [NaNO_3_ (120.0 g⋅L^–1^), KCl (10.4 g⋅L^–1^), MgSO_4_⋅7H_2_O (10.4 g⋅L^–1^) and KH_2_PO_4_ (30.4 g⋅L^–1^)] and 0.1% (v/v) trace elements solution [ZnSO_4_⋅7H_2_O (22.0 g⋅L^–1^), H_3_BO_3_ (11.0 g⋅L^–1^), MnCl_2_⋅4H_2_O (5.0 g⋅L^–1^), FeSO_4_⋅7H_2_O (5.0 g⋅L^–1^), CoCl_2_⋅6H_2_O (1.7 g⋅L^–1^), CuSO_4_⋅5H_2_O (1.6 g⋅L^–1^), Na_2_MoO_4_⋅2H_2_O (1.5 g⋅L^–1^) and Na_4_EDTA (50.0 g⋅L^–1^)]. The pH was adjusted to 6.5 with NaOH and the medium sterilized in an autoclave (15 min; 110°C).

*Neurospora crassa* was cultivated in liquid minimal medium containing K_2_PO_4_ (1 g⋅L^–1^) and glucose (10 g⋅L^–1^) dissolved in distilled water. The pH was adjusted to 7 with 10% phosphoric acid and the medium sterilized in an autoclave (10 min; 110°C). Filter sterilized salts solution [1% (v/v), *per* 100 mL: NaNO_3_ (30 g), MgSO_4_.7H_2_O (5 g), KCl (5 g), ZnSO_4_.H_2_O (100 mg), CuSO_4_.5H_2_O (50 mg), HCl 37% (10 μL) and FeSO_4_.7H_2_O (100 mg)] was added after autoclaving.

### Minimal Inhibitory Concentrations (MICs) of Ionic Liquids

The minimal inhibitory concentrations (MICs) were determined as described previously (Petkovic et al., 2010). Final concentrations of ionic liquids in growth media ranged from 100 μM up to maximum solubility. Each liquid medium (1 mL) was inoculated with 10^6^ spores and divided into four wells (0.2 mL each) of a 96 well microtiter plate. Cultures were incubated in the dark, at 30°C (*A. nidulans* and *N. crassa*) or 37°C (*A. fumigatus*) for 7 days. Fungal growth (or lack thereof) was determined at the end of incubation gauging by eye the formation of mycelium (turbidity). The lowest concentration that inhibited the formation of mycelium was defined as the MIC. Values should not be interpreted as absolute ones, but as an indication of the inhibitory and the fungicidal upper concentration limits.

### Metabolite Production

Fungal cultures (100 mL) were initiated from 10^6^ spores *per* mL in the respective minimal medium. Liquid cultures were incubated in the dark at 30°C (*N. crassa*, *A. nidulans*) or 37°C (*A. fumigatus*) with orbital agitation of 200 rpm. After 24 h, the ionic liquid supplement was added at 50% (i.e., 1.7 mM ChoDec for *A. fumigatus*) or 80% of the MIC (i.e., 0.96 M and 1.76 M ChoCl for *N. crassa* and *A. nidulans*, respectively, and 2.7 mM ChoDec for *A. fumigatus*). Negative conditions (without ionic liquid supplement) were prepared in parallel. Cultures were grown for 10 more days under agitation (100 rpm). At the end of incubation, the media supernatants were separated from mycelia using vacuum assisted filtration with miracloth (Merck Millipore Calbiochem). *Neurospora crassa* filtrates required the use of protease inhibitors (cOmplete Protease Inhibitor Cocktail, Waters) as preliminary tests showed degradation of untreated extracts (data not shown). The mycelia and filtrates were frozen immediately in liquid nitrogen and lyophilized.

### Metabolite Extraction

Lyophilized filtrates were homogenized in 10 mL Milli-Q water, extracted three times with ethyl acetate (1:1) and the combined ethyl acetate fractions dried under soft nitrogen flow. Peptide enrichment was achieved using the Sep-Pak plus C18 cartridge (Waters) as previously described (Krause et al., 2006). The samples were re-dissolved in 10 mL of MeOH/H_2_O (1/2, v/v) and loaded with a syringe into a conditioned cartridge. The cartridge was washed with 10 mL of Milli-Q water and 10 mL MeOH/H_2_O (1/2, v/v). The retained compounds were eluted with 10 mL of MeOH to a pre-weighed glass tube and dried under soft nitrogen flow; crude extracts. Conditioning of the cartridge was done successively with 10 mL of MeOH, Milli-Q water and MeOH/H_2_O (1/2, v/v).

### Chromatographic Analysis

The crude extracts in 10% (w/v) MeOH, were chromatographically separated using a Waters Acquity chromatographer with Photodiode Array detector, cooling auto-sampler and column oven. A Symmetry C18 column (250 × 4.6 mm), packed with end-capped particles (5 μm, pore size 100 Å) (Waters Corporation), was used at 26°C. Data were acquired using Empower 2 software, 2006 (Waters Corporation). Samples were injected using a 10 μL loop operated in full loop mode. The mobile phase, at a flow rate of 0.9 mL⋅min^–1^, consisted of a solution of 0.1% trifluoracetic acid in water (v/v) (TFA, solvent A) and Acetonitrile (ACN, solvent B), set to a linear gradient of 99.5 to 0% of solvent A during 30 min, followed by 100% of solvent B for 10 min, 2 min to return to the initial conditions, and additional 10 min to re-equilibrate the column. The chromatographic profiles of the samples were obtained at the wavelength of 205 nm. Sample fractionation was performed with a Fraction collector III (Waters) connected to the Acquity chromatographer (Waters) using the same conditions described above. The collected fractions were dried under nitrogen flow and kept at 4°C (short term) or −20°C (long term) until further analysis.

### Total Amino Acid Hydrolysis and Analysis

Total hydrolysis of the crude extracts (approximately 100 μg) was performed using 6 N HCl for 24 h at 110°C under inert atmosphere (nitrogen flushed). The fractions were also hydrolyzed for 1 h at 150°C under inert atmosphere (nitrogen/vacuum cycles) in a Workstation Pico-Tag (Waters). Hydrolyzed samples were further analyzed using the AccQTag Ultra Amino Acid Analysis Method (eluent concentrates, derivatization kit and standard mixture of amino acid hydrolyzates, Waters) (Penrose et al., 2001; Armenta et al., 2010). Briefly, the hydrolyzed samples, the standards of Aib and ACC, and the standard mixture of amino acid hydrolyzates were derivatized following the manufacturer’s instructions. The obtained derivatives were separated on an AccQTag Ultra column (100 mm × 2.1 mm, 1.7 μm) by reversed phase ultra-performance liquid chromatography (UPLC), and detected by fluorescence (FLR), according to the following details. The column heater was set at 55°C, and the mobile phase flow rate was maintained at 0.7 mL⋅min^–1^. Eluent A was 5% AccQTag Ultra concentrate solvent A and eluent B was 100% AccQTag Ultra solvent B. The separation gradient was 0–0.54 min (99.9% A), 5.74 min (90.9% A), 7.74 min (78.8% A), 8.04 min (40.4% A), 8.05–8.64 min (10.0% A) and 8.73–10.50 min (99.9% A). Two microliters (2 μL) of sample were injected for analysis using a 10 μL loop. The FLR detector was set at 266 and 473 nm of excitation and emission wavelengths, respectively. Data were acquired using Empower 2 software, 2006 (Waters). Calibration curves of each standard were used to quantify amino acids, the values are represented as the relative % of total amount of amino acids. The total area of peaks was used to determine the overall % of identification.

### Antibiotic Evaluation of Peptide-Based Metabolites

The extracts were assessed for their antimicrobial activity against gram-positive bacteria *Staphylococcus aureus* NCTC8325 and gram-negative bacteria *Escherichia coli* TOP 10, following the standard methodology implemented by the Clinical and Laboratory Standards Institute (Clinical and Laboratory Standards Institute [CLSI], 2018). First, bacteria were grown until approximately 1 to 2 × 10^8^ CFU⋅mL^–1^ in Mueller Hinton Broth (MHB, Panreac). Then, two-fold serial dilutions were performed to obtain final extracts concentrations between 1,000 and 62.5 μg⋅mL^–1^. Plates were incubated at 37°C for 24 h in a Bioscreen C analyzer (Oy Growth Curves Ab Ltd), taking hourly absorbance measurements (600 nm). All tests were done in triplicate; abiotic (medium alone) and biotic controls (each bacterium without extract) were included for each replicate.

After incubation with the crude extracts, 100 μL of each sample were mixed with 10 μL of 5 mg⋅mL^–1^ 3-(4,5-dimethylthiazol-2-yl)-2,5-diphenyl tetrazolium bromide (MTT) (Sigma Aldrich) in PBS (96-well microtiter plates) and incubated (dark, 37°C, 30 min). Then, 100 μL 10% SDS in 0.01 M HCl were added to each well and plates incubated for 2 h in the dark at room temperature. Absorbance was measured at 560 and 700 nm using Tecan Infinite 200 Microplate (Männedorf, Switzerland). For quantification, values at 560 nm were subtracted from the values at 700 nm. A second aliquot of 50 μL was used to label the cells with propidium iodide (20 μM PI, Biotium) and SYTO9 (3 μM; Alfagene) and further incubated for 15 min at room temperature with agitation. Fluorescence intensity was measured with a FLUOstar OPTIMA Microplate Reader (BMG⋅Labtech) using a 488/20 nm excitation filter (for both SYTO9 and PI), and a 528/20 nm (SYTO9 emission wavelength) and 645/40 nm (PI emission wavelength) emission filter. The signal from the staining solution (SYTO9/PI) was subtracted from all data to minimize cross-signal background. Microscopy assessment of the live/dead staining was done on a Leica DM 6000B upright microscope equipped with an Andor iXon 885 EMCCD camera and controlled with the MetaMorph V5.8 software, using the 100 × 1.4 NA oil immersion objective plus a 1.6× optovar, the fluorescence filter sets FTIC + TX2 and Contrast Phase optics. Images were analyzed by FIJI software (Fiji Is Just ImageJ). IC_50_ (half maximal inhibitory concentration of a compound) values were calculated from dose response curves constructed by plotting cell viability (MTT data) versus extract concentration (μg⋅mL^–1^) using the Logit regression model (dose effect analysis tool of XLSTAT).

### LC-MS/MS Analysis

NanoLC-MS/MS analysis was performed using an Eksigent Nano-LC 425 System (Eksigent, SCIEX) coupled TripleTOF 6600 + mass spectrometer (SCIEX). Samples (<1 μg⋅mL^–1^; 4 μL each) were analyzed as follows. *N. crassa* samples were loaded on a C18 PepMap trap column (5 μm, 300 μm × 5 mm) (Thermo Scientific) at a flow rate of 2 μL min^–1^ for 10 min using 2% (v/v) ACN + 0.05% (v/v) TFA as mobile phase (Ribeiro et al., 2020); then peptides were separated at a flow rate of 300 nL⋅min^–1^ into a C18 PepMap 100 column (75 μm × 150 mm, 3 μm, 100 Å) (Thermo Scientific) using a linear binary gradient of 0.1% formic acid (v/v) in water (solvent A) and 0.1% formic acid (v/v) in ACN (solvent B) for a total running time of 100 min. Gradient program was 3–60% B in 60 min, then 40% B from 60 to 70 min, increasing again to 80% B to wash the column and finally re-equilibrating to the initial conditions (3% B) for 20 min. For *A. fumigatus* samples, the initial step of pre-concentration was the same as for *N. crassa*. Running gradient was different and adapted from Marik et al. (2018). Briefly, samples were separated at a flow rate of 300 nL⋅min^–1^ using a linear gradient of 0.05% (v/v) TFA in water (Solvent A) and 0.05% (v/v) TFA in ACN/MeOH (1:1, v/v) (solvent B). Gradient program for solvent B was 65% for 5 min, 65–80% from 5 to 45 min, then 100% until 75 min and last 65% from 76 to 81 min. MS data was acquired in positive mode over a mass range 300–1,250 m/z (for *N. crassa*) and 100–2,000 m/z (for *A. fumigatus*), with 250 ms of accumulation time. The 30 most intense ions were selected to perform fragmentation with high sensitivity mode using the automatically adjusted system of rolling collision energy. MS/MS scans were acquired over a mass range 100–1500 m/z with an accumulation time set at 50 ms; raw data files.

### Molecular Networking and Compound Dereplication Using GNPS Platform

Raw data files (.wiff) were converted to open format mzXML using ProteoWizard MSConvert version 3.0.10051 (Kessner et al., 2008) to transform spectra from profile to centroid mode.^1^ Data files were uploaded on GNPS through WinSCP (version 5.17.3) to generate a molecular network according to guidelines (Aron et al., 2020), which can be openly accessed.^2^ To create the network, first all MS/MS spectra were aligned. Data were then filtered by removing all MS/MS peaks within ± 17 Da of the precursor m/z. MS/MS spectra were window filtered by choosing only the top six peaks in the ± 50 Da window throughout the spectrum. The precursor ion mass tolerance was set to 2.0 Da and a MS/MS fragment ion tolerance of 0.5 Da. A network was then created where edges were filtered to have a cosine score >0.7 and more than 6 matched peaks. Cosine score ranges from 0 (different parent ions) to 1 (structurally similar compounds) (Watrous et al., 2012). Edges between two nodes were kept in the network only if each of the nodes appeared in each other’s respective top 10 most similar nodes. The maximum size of a molecular family was set to 100, and the lowest scoring edges were removed until the size was below this threshold. Self-loop nodes indicate that there is no structurally related molecule present in the sample. The spectra in the network were then searched against GNPS’ spectral libraries (e.g., MassBank, ReSpect, and NIST) to assign a putative identification (Wang et al., 2016). The library spectra were filtered in the same manner as the input data. All matches kept between network spectra and library spectra were again required to have a score >0.7 and at least 6 matched peaks. The resulting molecular network was visualized using Cytoscape software v3.7.2 (Shannon et al., 2003). The molecular network is comprised by nodes (specific consensus spectrum) connected with edges (significant pairwise alignment between nodes). Nodes were labeled with putative identification and colored according to the group where the precursor was detected; edges thickness is proportional to cosine score. Complementary to library matching, DEREPLICATOR + workflow allow to predict fragmentation spectra *in silico* from known structures and to search for candidate structures in chemical databases (Mohimani et al., 2018). MS/MS data were used as input. The output table with potential candidates was integrated into the molecular network using Cytoscape. Manual validation of putative identifications was done through removal of hits from negative mode MS (not acquired herein) or after mirror plots (library compounds vs. input spectra) inspection. According to Sumner et al. (2007), putative annotations of compound and molecular families based on GNPS correspond to level 2 (Sumner et al., 2007). Herein, no standards were used to validate identifications. Complementary analysis of the MS spectra of the fractions was done using the NRPro tool^3^ which includes databases not represented in GNPS, namely NORINE and NPAtlas (Ricart et al., 2020). Input data (MS/MS spectra in .mgf format) were uploaded, and search parameters were set as follows: peptide tolerance of 0.02 Da and fragment mass tolerance of 0.01 Da with M + H ionization with a charge up to 2. Decoy was activated; generates *p*-values associated with the identifications. Hits were validated (*p*-value < 0.05) upon further inspection of the number of scored peaks vs. annotated peaks.

### Statistical Analysis

Data were analyzed using standard statistical software (Origin v8.5 Software, San Diego, CA, United States, and GraphPad Software Prism v7, San Diego, CA, United States). Three biological replicates were executed. Results are expressed as mean value ± standard deviation. The statistical significance of values between conditions was evaluated by One-Way ANOVA test. Differences were considered significant when the *p*-value ≤0.05.

## Results and Discussion

### Ionic Liquid Supplements Triggered a Metabolic Shift in the Fungal Cultures

It has been observed that culture conditions greatly impact secondary metabolism (Mathew Valayil, 2016). This explains the rationale behind the OSMAC approach to alter secondary metabolism in fungi (Chiang et al., 2009), and the usage of ionic liquids supplements as well (Petkovic et al., 2009; Alves et al., 2016). In the present study, two choline based ionic liquids were chosen, namely ChoCl and ChoDec. The first one has been previously reported to boost differential metabolic responses in fungi (Martins et al., 2013; Alves et al., 2016). ChoDec because longer alkyl chains in the anion have higher toxicity toward fungi and accordingly, less amounts are needed to induce stress (Petkovic et al., 2010; Hartmann et al., 2015). The MIC values for each fungus – *A. nidulans*, *A. fumigatus*, and *N. crassa* – are listed in Table 1. Choline based ionic liquids have been shown to be biodegradable, specifically the choline cation was observed to be partially degraded after 15 days of incubation with either *A. nidulans* and *N. crassa* (Martins et al., 2013). The decanoate anion was herein undetectable in the medium supernatant (chromatographic analysis) after 5 days of incubation (data not shown). Similar degradation yields have been previously reported for other filamentous fungi (Boethling et al., 2007; Petkovic et al., 2010).

**TABLE 1:** Minimal inhibitory concentrations of the cholinium-based ionic liquids (choline chloride, ChoCl and choline decanoate, ChoDec) used as media supplements for each fungal strain.

	ChoCl [M]	ChoDec [mM]
*A. fumigatus*	1.7	3.4
*A. nidulans*	2.2	2.6
*N. crassa*	1.2	–

**FIGURE 1:**
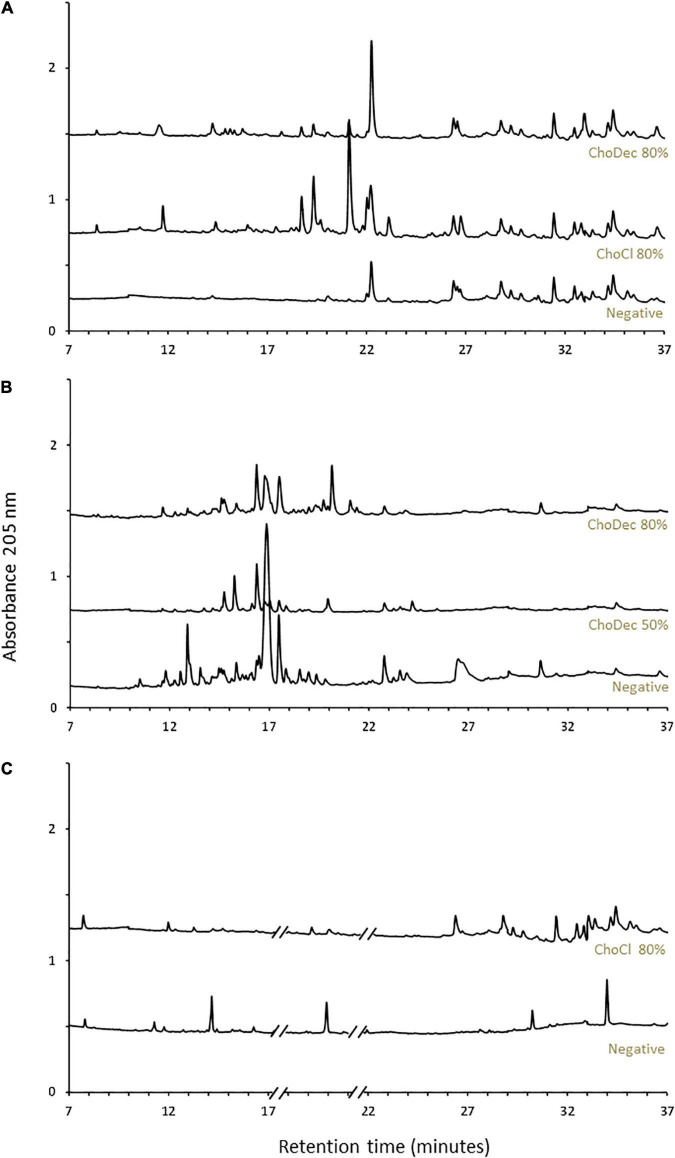
Ionic liquid supplements triggered a metabolic shift in the fungal cultures. Chromatographic analyses of the metabolic footprint of *A. nidulans* **(A)**, *A. fumigatus* **(B)**, and *N. crassa* **(C)** crude extracts. Crude extracts are from cultures grown for 10 days in either choline chloride (ChoCl) or choline decanoate (ChoDec) supplemented media, at 50 or 80% of the MIC, and from cultures without ionic liquid supplementation (i.e., negative controls). Truncated parts of the chromatogram from *N. crassa* cultures **(C)** correspond to the elution of protease inhibitors. The *y*-axis scale represents the base peak intensity, where units are arbitrary.

Upon 10 days of incubation, fungal cultures were harvested, and the cultivation media were extracted. Secondary metabolites were enriched by liquid-liquid extraction with ethyl acetate, followed by solid-phase extraction resulting in peptide enriched fractions (Krause et al., 2006). The metabolic footprints (i.e., pool of metabolites produced at a given point under certain culture conditions) of the crude extracts were investigated by liquid chromatography (Figure 1). *A. nidulans* and *A. fumigatus*, in contrast to *N. crassa*, show high basal diversity of metabolites. In general, the profiles are distinct in cultures grown in the supplemented media compared to the negative control (without supplementation). The observed metabolic footprints depend on the ionic liquid supplement (Figure 1A) and of its concentration as well (*viz*, 50 and 80% of the MIC of ChoDec) (Figure 1B). This result corroborates preceding observations that distinct ionic liquids induced distinct metabolic alterations on the fungal metabolism, increasing, in general, the diversity of synthesized low molecular-weight molecules (Petkovic et al., 2009; Martins et al., 2013; Alves et al., 2016). Using a similar approach, monodictyphenone and orsellinic acid, otherwise cryptic metabolites, accumulated (in a pool of *ca.* 40 ion masses) in cultures of *A. nidulans* grown in medium supplemented with 1-ethyl-3-methylimidazolium chloride (Alves et al., 2016). Orsellinic acid had been also identified in *A. nidulans* during co-cultivation with *Streptomyces* spp. (Fischer et al., 2018). Proteomic analyses of *A. nidulans* and *N. crassa* cultures, showed that several biological processes and pathways were affected upon supplementation with ChoCl, provoking also an accumulation of stress-responsive proteins and osmolytes (Martins et al., 2013).

### Total Amino Acid Hydrolysis Discloses the Presence of Non-proteinogenic Residues in *Neurospora crassa* and *Aspergillus fumigatus* Extracts

Fungi are able to use both proteogenic and non-proteinogenic amino acids (NPAAs) for incorporation in NRPs; NPAAs may positively impact the stability, potency, permeability, oral bioavailability, and immunogenicity of peptides as they do not occur naturally in humans (Ding et al., 2020). In fact, an important feature of many fungal antimicrobial peptides is the presence of NPAAs or other α-hydroxy and carboxylic acids (Mootz et al., 2002). A previous study has shown that ChoCl supplementation of *N. crassa* growth medium led to the increased expression of the 1-aminocyclopropane-1-carboxylate (ACC) deaminase, which mediates the formation of ACC (Martins et al., 2013). In some fungi, the presence of ACC has been linked to the peptaibiotics neofrapeptins and acretocins, isolated from *Geotrichum candidum* SID 22780 and *Acremonium crotocinigenum* cultures, respectively (Fredenhagen et al., 2006; Brückner et al., 2019). Peptaibiotics show a unique structure varying from 5 to 21 amino acid residues, including numerous NPAAs, mainly α-aminoisobutyric acid (Aib), and/or lipoamino acids (Degenkolb et al., 2003; Degenkolb and Brückner, 2008). Aib has been found to correlate to specific types of secondary structures, namely helical structures, improving peptide functioning and increasing enzymatic resistance (Niu et al., 2020).

**FIGURE 2:**
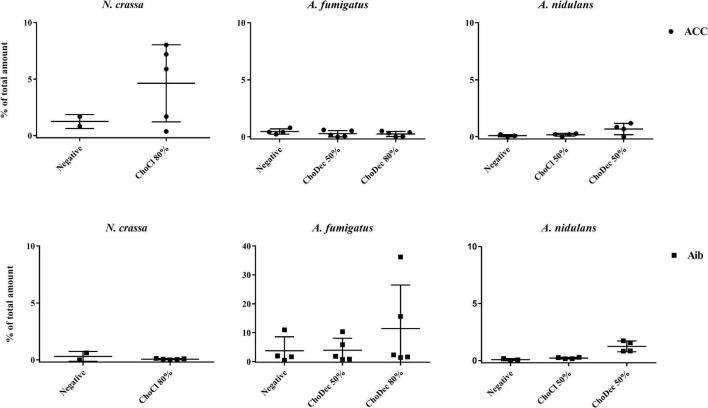
Total amino acid hydrolysis discloses the presence of two non-proteinogenic residues in *N. crassa* and *A. fumigatus* extracts. Scatter plot depicting individual values of percentage (%) of total amount of 1-aminocyclopropane-1-carboxylate, ACC (●) and α-aminoisobutyric acid, Aib (■) obtained from total hydrolysis of the crude extracts derived from cultures grown in media with or without (i.e., negative) supplementation. The *y*-axis scales are not normalized to allow easier visualization of the amount of either non-proteinogenic amino acid in each condition.

To verify if the ionic liquid-supplements have induced the production of peptides containing NPAAs, specifically ACC and Aib, the total amino acid content of extracts (upon hydrolysis) were chromatographically analyzed. Both NPAAs could be detected, most evident in *N. crassa* and *A. fumigatus* (Figure 2). Specifically, in *N. crassa* ACC levels show increasing trend upon ChoCl supplementation, consistent with the accumulated levels of ACC deaminase described before (Martins et al., 2013). *A. fumigatus* control extracts show low levels of Aib with a slight, but not statistically significant, increase when the culture is supplemented with ChoDec (at 80% of MIC). In *A. nidulans*, an increasing trend in either NPAAs upon ChoDec supplementation was observed, but the overall amounts of Aib and ACC are substantially lower compared to the other two fungi.

Ionic liquid-exposure altered the pattern of the overall amino acid content, suggestive of an altered peptidome profile (Supplementary Table 1). Nonetheless, no meaningful alterations were found (pair-wise ANOVA) in the detected amounts of each amino acid with or without media supplementation, possibly consequence of high variability between the biological replicates. For *A. fumigatus* around 30% and for *N. crassa* 45–65% of the peaks could not be assigned to any of the amino acid standards. For *A. nidulans*, the values were lower: 4–7% (negative and ChoCl supplemented extracts) and 27% (ChoDec supplemented extracts). Despite these inherent technical fragilities, this analysis provides an estimation of the amino acid profiles of each sample, and excitingly point to the existence of peptides containing ACC and/or Aib in either crude extract from ionic liquid supplemented cultures. Based on these results, *N. crassa* and *A. fumigatus* extracts were selected for subsequent analyses focusing antibacterial efficacy and compositional signature (LC-MS/MS).

### *Neurospora crassa* and *Aspergillus fumigatus* Crude Extracts Depict Antibacterial Activity

**FIGURE 3:**
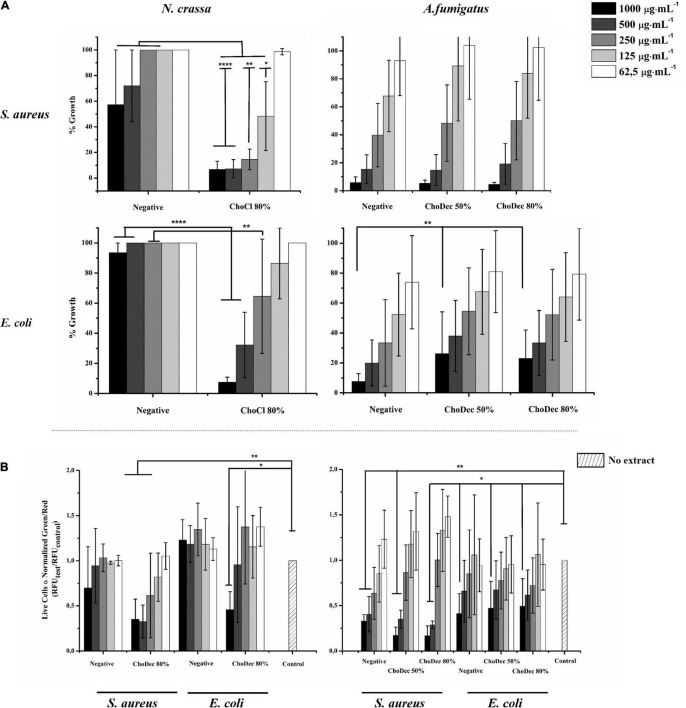
*N. crassa* and *A. fumigatus* crude extracts depict antibacterial activity against *S. aureus* and *E. coli*. Cell viability measured by MTT assay of extracts derived from cultures grown in media with or without (i.e., negative) supplementation **(A)**. Quantification of bacterial viability through the normalized green/red ratio (i.e., SYTO9 (green, live cells)/PI (red, dead cells) staining) **(B)**. Statistically significant differences are depicted; *p ≤ 0.05, **p ≤ 0.01, ****p ≤ 0.0001.

**FIGURE 4:**
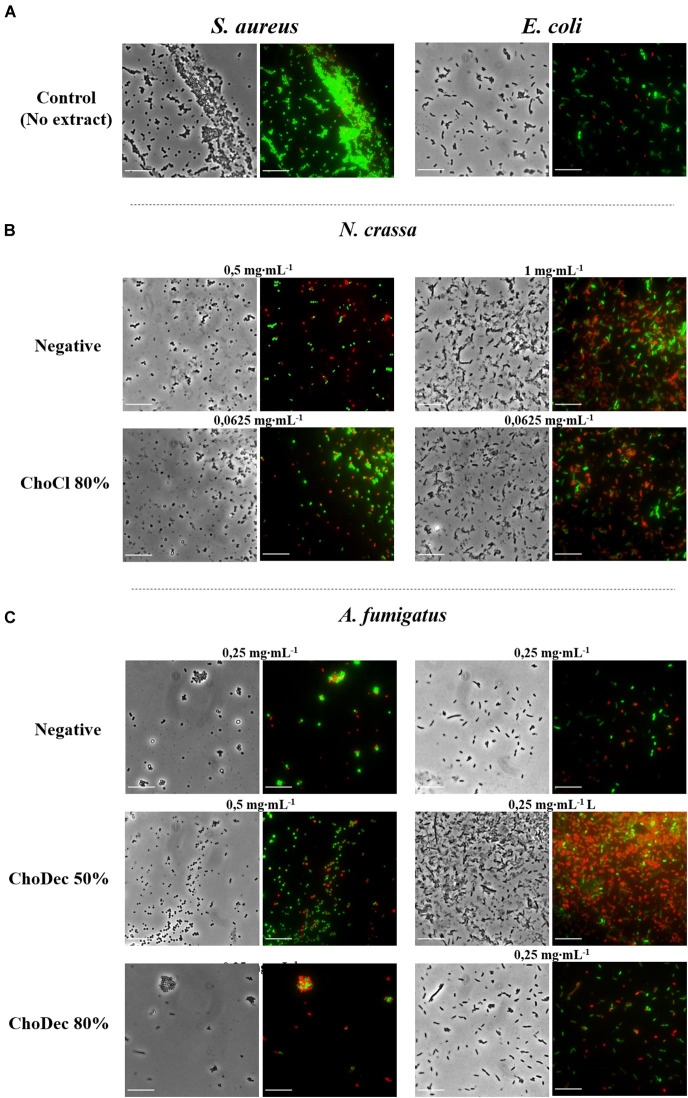
*N. crassa* and *A. fumigatus* crude extracts led to significant lysis of *S. aureus* and *E. coli* cells, which is denoted by the red labeling. Microscopic snapshots of *E. coli* and *S. aureus* grown in the absence of extract **(A)** and in the presence of crude extracts derived from cultures grown in media with or without (*i.e.*, negative) supplementation: *N. crassa* **(B)** and *A. fumigatus* **(C)**. Images of bacteria at concentrations near the measured IC_50_ for each crude extract are shown. Cells were stained with SYTO9 (green) and PI (red) denoting live and dead cells, respectively. Scale bar, 10 μm.

**FIGURE 5:**
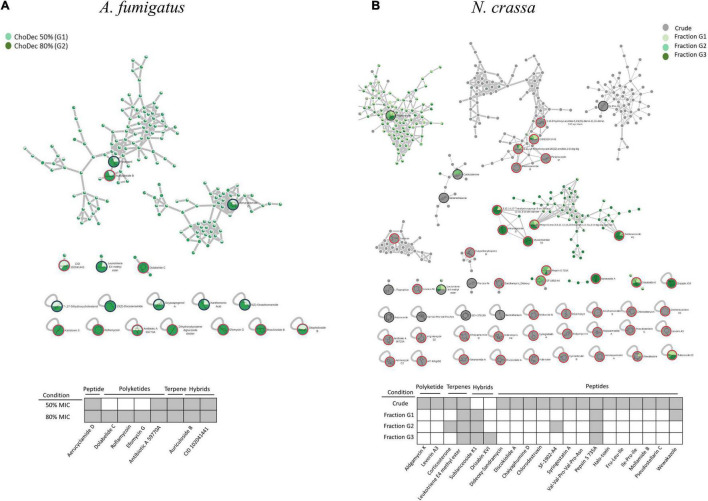
The molecular network generated by the GNPS tool using the MS data acquired for *A. fumigatus* **(A)** and *N. crassa* **(B)** extracts derived from ionic liquid supplemented cultures, considering the putatively identified compounds the accumulation, among other metabolites of macrolides and peptides, respectively. Putative identifications retrieved by using molecular networking analysis and compound dereplication in GNPS, from spectral match (*) and *in silico* tool DEREPLICATOR + . For *N. crassa*, isolated fractions (G1, G2, and G3) as well as crude (G4) extracts were analyzed; whereas for *A. fumigatus*, extracts from cultures supplemented with choline decanoate at 50% (G1) and 80% (G2) of the MIC. Only peaks with signal intensity >1.5⋅10^7^ (for *A. fumigatus*: G1 and G2) and >3⋅10^7^ (for *N. crassa*: G4) in the total ion chromatogram are depicted (Full list in Supplementary Table 3). For *N. crassa* fractions, G1 to G3, the two most intense peaks in the total ion chromatogram are depicted.

The antibacterial activity of *N. crassa* and *A. fumigatus* extracts against *S. aureus* and *E. coli* was assessed using the broth dilution method. For each crude extract, two-fold dilutions of an initial concentration of 1,000 μg⋅mL^–1^ were performed. Bacterial growth, inferred by the medium turbidity (600 nm), was measured for 24 h. After growth, bacterial viability was evaluated via measurements of the metabolic activity (MTT) and the live/dead cell ratio obtained from fluorescent staining quantifications. After 24 h, cell viability decreased significantly relative to the bacterial control, reflected in the MTT and live/dead cell ratio quantifications (Figure 3 and Supplementary Table 2). Microscopic snapshots show major cell lysis upon exposure to extracts derived from ionic liquid-supplemented cultures compared to the bacterial control (no extract) (Figure 4). Based on the estimated IC_50_ values (Table 2), the supplementation compared to control conditions, increased greatly the bactericidal activity of the derived *N. crassa* extracts, but not those of *A. fumigatus*. At this stage, the observed activity cannot be linked to a specific compound. To pinpoint potential candidates, untargeted MS analyses using the GNPS platform were applied. A total of 52 and 18 compounds were identified in *N. crassa* and *A. fumigatus* extracts derived from the ionic-liquid supplemented cultures, respectively (Figure 5 and Supplementary Tables 3, 4). By eliminating compounds of low signal intensity, the most promising candidates potentially produced by *A. fumigatus* are macrolides and terpenes, whereas for *N. crassa* are cyclic peptides, including five depsipeptides; structurally of high pharmacological interest (Table 3). Fractionation of the later, added another cyclic peptide to the pool of compounds annotated through the GNPS tool; likely of low abundance in the crude extract. Analysis of their whole chemical landscape highlighted, however, a weak sample deconvolution with many compounds present in the three fractions. Through their direct query in the NRPro database, five additional hits of cyclic peptides (including one depsipeptide) were found (Supplementary Table 5).

**TABLE 2:** IC_50_ values determined for *A. fumigatus* and *N. crassa* crude extracts from media supplemented or not (negative control) with choline chloride (ChoCl) or choline decanoate (ChoDec), at 50 or 80% of the MICs.

Fungal strain	Bacterial strain	Extract tested	IC_50_ (μg⋅mL^–1^)
*N. crassa*	*E. coli*	Negative	1,280
		ChoCl 80%	103
	*S. aureus*	Negative	310
		ChoCl 80%	70
*A. fumigatus*	*E. coli*	Negative	120
		ChoDec 50%	310
		ChoDec 80%	350
	*S. aureus*	Negative	310
		ChoDec 50%	260
		ChoDec 80%	470

*IC_50_ represents the crude extract concentration that inhibits bacterial activity by 50% and were calculated from curves constructed by plotting cell viability (MTT data) vs. extract concentration (μg⋅mL^–1^).*

**TABLE 3:** Untargeted LC-MS/MS analyses of *A. fumigatus* and *N. crassa* extracts derived from ionic liquid supplemented cultures suggests the accumulation, among other metabolites of macrolides and peptides, respectively.

Putative identification	Exact mass	Condition	Class	Reported activity	References
***Aspergillus fumigatus***					
Dolabelide C	796.497	G2	Macrolide	Antitumor	Suenaga et al., 1997
Roflamycoin	738.455	G2	Macrolide	Antifungal; antiprotozoalc	Schlegel and Thrum, 1971; Han et al., 2021
Efomycin G	1010.58	G2	Macrolide	Antibacterial; antitumor	Wu et al., 2013; Supong et al., 2016; Gui et al., 2019
Antibiotic A 59770A	1000.63	G1, G2	Macrolide	Pesticidal agents	Hoehn et al., 1990
Aerucyclamide D*	603.06	G1, G2	Cyclic peptide	Antiparasitic	Portmann et al., 2008
7α,27-Dihydroxycholesterol*	401.342	G1, G2	Steroid	Not reported	Brown and Jessup, 1999
Auriculoside B	1214.64	G1, G2	Pregnane glycoside	Antitumor	Zhang et al.
CID 102041441	810.477	G1, G2	Pregnane glycoside	Not reported	Deng et al., 2010
***Neurospora crassa***					
Levorin A3	1092.58	G4	Macrolide	Antifungal	Pawlak et al., 2005; Szczeblewski et al., 2017
Dideoxy-Sandramycin	1188.56	G4	Cyclic depsipeptide	Antitumor	Boger and Chen, 1997
Discokiolide A	1026.51		Cyclic depsipeptide	Antitumor	Tada et al., 1992
Chaiyaphumine D	644.296	G4	Cyclic depsipeptide	Not reported	Grundmann et al., 2014
Chlorodestruxin	629.319	G4	Cyclic depsipeptide	Anti-insecticidal	Gupta et al., 1989
SF-1902-A4	667.452	G2, G4	Cyclic lipodepsipeptide	Antibacterial	
Syringostatin A	1178.59	G4	Cyclic lipodepsipeptide	Antifungal	Sorensen et al., 1996
Val-Val-Pro-Val-Pro-Asn*	651.396	G4	Peptide	Not reported	In-house library from GNPS
Pepsin S 735A	685.463	All samples	Peptide	Protease inhibitor	Morishima et al., 1970; OMURA et al., 1986
Halo-toxin	626.343	G4	Peptide	Not reported	Kajimoto et al., 1989
Fru-Leu-Ile*	407.239		Peptide	Not reported	In-house library from GNPS
Ile-Pro-Ile*	342.239	G4	Peptide	Not reported	In-house library from GNPS
Mollamide B	696.367	G4	Cyclic peptide	Antimalarial, antivirus, antitumor	Donia et al., 2008
Pseudostellarin C	812.443	G4	Cyclic peptide	Tyrosinase inhibitor; antitumor	Morita et al., 1994
Wewakazole	1140.54	G1, G4	Cyclic peptide	Antitumor	Nogle et al., 2003; Gogineni and Hamann, 2018
Corticosterone*	347.222	G2, G4	Terpene	Not reported	Steiger and Reichstein, 1938
Leukotriene E4 methyl ester*	459.22	All samples	Lipid	Immunomodulation	Cohen et al., 2002
Orizabin XIV	1120.6	G3	Glycolipid	Antitumor; β-1-3-glucan synthase inhibitor; antibacterial	Pereda-Miranda and Hernández-Carlos, 2002
Sublanceoside K1	1082.57	G1, G2, G3	Terpene glycoside	Not reported	Warashina and Noro, 2006

Putative identifications retrieved by using molecular networking analysis and compound dereplication in GNPS, from spectral match (*) and in silico tool DEREPLICATOR+. For N. crassa, isolated fractions (G1, G2, and G3) as well as crude (G4) extracts were analyzed; whereas for A. fumigatus, extracts from cultures supplemented with choline decanoate at 50% (G1) and 80% (G2) of the MIC. Only peaks with signal intensity >1.5⋅10^7^ (for A. fumigatus: G1 and G2) and >3⋅10^7^ (for N. crassa: G4) in the total ion chromatogram are depicted (Full list in Supplementary Table 3). For N. crassa fractions, G1 to G3, the two most intense peaks in the total ion chromatogram are depicted.

The results show the production of antimicrobial compounds in *N. crassa* cultures under ionic liquid supplementation, likely associated to production of metabolites otherwise cryptic. The hypothesis that these antimicrobial compounds support *N. crassa* competitiveness in specific niches deserves further consideration. However, contrary to that observed for *N. crassa*, the supplementation did not increase the antibacterial activity of *A. fumigatus* derived extracts. Regardless of these contrasting results, the chemical landscape of either extract was further analyzed using an untargeted MS metabolomics approach.

### LC-MS/MS Analyses of *Aspergillus fumigatus* Extracts Derived From Ionic Liquid Supplemented Cultures, Suggests the Accumulation of Macrolides, Among Other Metabolites

The MS spectra collected for the *A. fumigatus* extracts derived from the ionic liquid supplemented cultures were subjected to a molecular networking analysis on the web-based platform GNPS. This platform relies on the principle that structurally similar compounds will have similar MS/MS fragmentation patterns, and hence allows deconvolution of large MS datasets, annotation, and discovery of novel and/or analog compounds. This automated annotation belongs to a class 2 classification (Sumner et al., 2007), therefore all compounds identification discussed below remain putative, requiring, for targeted compounds, further validation in the near future.

The metabolic footprints of *A. fumigatus* extracts grown in media supplemented with 50% (G1) or 80% (G2) of the ChoDec MIC concentration were analyzed. In this case, of 1471 nodes, 684 nodes were clustered into 135 molecular families and the remaining 787 did not share any connection (full dataset hyperlink in Supplementary Table 3). In total, 18 metabolites were putatively identified, 9 by spectral match in GNPS databases (black border nodes) and 9 by *in silico* DEREPLICATOR + tool (red border nodes) (Figure 5A, full list in Supplementary Table 4). Most of the nodes correspond to metabolites produced in both conditions. Only compounds with signal intensity >1.5⋅10^7^ in the total ion chromatogram (with a clear separation from baseline values) will be discussed in greater detail (Table 3, bottom panel in Figure 5A). Half of these compounds belong to the class of polyketides, some of which were found only in G2 (80% MIC). In either sample, G1 and G2, the most frequently found polyketide compounds are macrolides; class of antibiotics composed of a large lactone ring with a sugar attached. The macrolides putatively identified were dolabelide C, efomycin G, roflamycoin, and antibiotic A 59770A. The first has been reported in a sea hare (Suenaga et al., 1997), while the last three are known as bacterial metabolites (Schlegel et al., 1981; Hoehn et al., 1990; Klassen et al., 2019). Macrolides production in fungi has been, however, reported before; e.g., phaeospelide A in *Aspergillus oryzae* (Morishita et al., 2019). These extracts showed a more pronounced effect over *S. aureus* (Figure 3), consistent with the putative identification of macrolides. This class of compounds is usually bacteriostatic, most efficient against Gram-positive bacteria but can also be active against several Gram-negative bacteria (Arslan, 2022). In particular, efomycin is active against a number of drug-resistant pathogens (e.g., methicillin-resistant *S. aureus*) (Wu et al., 2013), and roflamycoin exerts activity against a broad spectrum of organisms (Han et al., 2021).

Apart from macrolides, in either sample, 7α,27-dihydroxycholesterol was identified, which belongs to the terpen(oid) class. It derives from cholesterol, and has been reported before in *A. fumigatus* metabolome (Gil-De-la-fuente et al., 2021). It is functionally relevant, helping the fungus to bypass the effects of ergosterol inhibitor class of antifungals (Xiong et al., 2005); a potential new drug target. Finally, PKs-terpenes hybrids (Keller, 2019), namely two pregnane glycosides were identified in either extract. They show broad spectrum activity (e.g., anticancer, analgesic, anti-inflammatory and antimicrobial) and to date only few have been reported in fungi, for example in *Aspergillus versicolor* cultures grown in rich medium for 15 days (Ding et al., 2019) and *Cladosporium* sp. grown in rice-based medium for 45 days (Yu et al., 2018). A single peptide was putatively identified, namely the cyclohexapeptide aerucyclamide D, a ribosomal metabolite that has been previous described in a cyanobacterium as a new antiparasitic compound (Portmann et al., 2008).

### LC-MS/MS Analyses of *Neurospora crassa* Extracts Derived From Ionic Liquid Supplemented Cultures, Suggests the Accumulation of Several Cyclic (Depsi)peptides, Among Other Metabolites

*Neurospora crassa* extracts derived from ChoCl supplemented cultures were chromatographically fractionated at the retention times of 15.6, 17.3, 19.6 min, corresponding to G1, G2, and G3, respectively. The peptidome of each fraction was analyzed as previously described (including the NPAAs ACC and Aib) (Supplementary Figure 1). G1 contains ACC; G2 contains Aib and ACC, and G3 contains none. Accordingly, G2 might comprise peptaibiotics. To determine the complete amino acid sequence of these fractions, Edman sequencing was attempted but failed, possibly due to a blocked N-terminal (Mootz et al., 2002). Overall, these observations further support the hypothesis that growth medium supplementation with ChoCl triggered production of peptaibiotics in *N. crassa*, otherwise cryptic.

The chemical landscape of these three samples and of the corresponding crude extract (G4) were analyzed, similarly to that done for *A. fumigatus*. A total of 5,249 nodes were obtained, 1,514 nodes clustered into 247 molecular families, and the remaining are self-loop nodes (full dataset hyperlink in Supplementary Table 3). To simplify, only clusters with putative hits are shown. In total, 10 compounds were putatively identified by comparison against GNPS databases (black border nodes) and 42 compounds by using the DEREPLICATOR + tool (red border nodes) (Figure 5B, full list in Supplementary Table 4). To focus the discussion, for G4 only the compounds presenting signal intensity >3.0⋅10^7^ in the total ion chromatogram are further considered, whereas for G1–G3 the two highest intensity signals are detailed if absent in G4 (Table 3, bottom panel in Figure 5B). G4 shows, as expected, the highest diversity of compounds. Similar to that found in *A. fumigatus* extracts, macrolides were the only polyketide compounds identified, specifically aldgamycin K and levorin A3. A single terpene, corticosterone, and one lipid-based metabolite, leukotriene E4 methyl ester, were putatively identified as well. Leukotrienes are eicosanoids produced by pathogenic fungi, suggested to act as virulence factors (Noverr et al., 2002). They are a subset of oxylipins, a class of metabolites that act mainly as lipid mediators, signaling spore development, metabolites production and virulence in fungi (Tsitsigiannis and Keller, 2007).

Remarkably, *N. crassa* seems to be an abundant producer of NRP, including peptides (linear and cyclic, 7 distinct compounds) and depsipeptides (5 distinct compounds) when grown in medium supplemented with ChoCl. Specially, two cyclic peptides were identified: pseudostellarin C and mollamide B, and five linear peptides: pepsin S 735A, halo-toxin, two tripeptides (Fru-Leu-Ile and Ile-Pro-Ile) and one hexapeptide (Val-Val-Pro-Val-Pro-Asn). Pepsin is the only linear peptide identified in all samples, possibly an artifact of the protease inhibitors herein used. The tri/hexapeptides identified here have never been reported before, questioning if these compounds are hydrolyzed products or are precursors of larger peptides. Besides, four cyclic depsipeptides were also putatively identified: discokiolide A, dideoxy-sandramycin, chlorodestruxin and chaiyaphumine D, all of which, expect the last, have been reported before and related to either antitumor or anti-insecticidal activities. Syringostatin A, a lipodepsinonapeptide, reported antifungal activity (Sorensen et al., 1996). In cyclic depsipeptides at least one amino acid is replaced by a hydroxylated carboxylic acid (α-hydroxy acid), resulting in a mix of amide and ester bonds in the core ring, conferring high stability (Taevernier et al., 2017; Wang et al., 2018). α-Hydroxy acids structural similarity to α-amino acids, ensures that depsipeptides can interact with numerous proteins yet showing higher resistance against hydrolyzing enzymes due to cyclization (Gentilucci et al., 2010; Stone and Deber, 2017). The higher resistance is expected to result in enhanced oral bioavailability (Sivanathan and Scherkenbeck, 2014). Several known depsipeptides contain NPAAs, for example 2-hydroxy-3-methyl-pentanoic acid, tiglic acid, α-aminobutyric acid, picolinic acid; constituents of compounds putatively identified in the extracts yet below the defined threshold of peak intensity, e.g., SCH-378199 and virginiamycin S5 (Supplementary Table 4). This observation is consistent with the presence of many non-identified amino acids in the *N. crassa* extracts (nearly half of the chromatographic peak area could not be assigned, Supplementary Table 1). The presence of Aib in this class of compounds remains to be seen. On the contrary, ACC is known to be a building block of depsipeptides, for example of BZR-cotoxin II, a metabolite of *Bipolaris zeicola*, and of CBS 154-94A, a metabolite of *Streptomyces* sp. (Fredenhagen et al., 2006). The last has antibiotic activity, acting as protein farnesyl transferase inhibitor. Finally, the cyclic lipodepsipeptide SF-1902-A4 was also identified (present also in G2); previous reported as antibacterial (Omoto et al., 1981). As above mentioned, most compounds were only found in the crude extract, except in the denoted cases. Looking to the two most intense peaks of G1–G3 fractions revealed the presence of wewakazole (G1, also in G4 but below the defined intensity threshold), orizabin XIV (G3), and sublanceoside K1 (in all fractions). The first compound, a cyclic dodecapeptide, has been reported to exhibit cytotoxicity against H460 human lung cancer cell line (Gogineni and Hamann, 2018). The second, is a glycolipid that inhibits the activity of 1,3-β-glucan synthase, required for cell wall synthesis in fungi (Castelli et al., 2002); a target of clinically approved antifungal drugs (Lima et al., 2019). The last, a terpene glucoside, has no reported bioactivity to date. Apart from these compounds, the remaining hits correspond to clusters containing spectra from all samples (G1 to G3), and include compounds belonging to resin glycosides, fatty acids, terpenoids and cyclic peptides. The chromatographic elution of the fractions (with close retention times) did not result in a clean separation, explaining why these clustered together in the molecular network. Since that NRPro tool (see text footnote 3) that is specific for NRPs is not included in the GNPS platform, the MS/MS spectra of the fractions were also queried in this database. Putative identifications were found only for G1 and G2 (Supplementary Figure 2 and Supplementary Table 5) revealing five additional cyclic peptides candidates. Specifically, G1 showed matches to guangomide A (depsipeptide), arbumelin and a cyclohexapeptide. The first two compounds have been previously identified in fungal strains, namely in *Trichothecium sympodiale* (Sy-Cordero et al., 2011) and *Calcarisporium arbuscular* (upon target inactivation of H3 deacetylase) (Mao et al., 2015), respectively. In G2, cyclotheonamide E3 and nostophycin were found, compounds identified before in a marine sponge (Nakao et al., 1998) and in a cyanobacterium (Fujii et al., 1999), respectively. In addition, the fractions were analyzed by NMR but their chemical complexity and low abundance of each constituent of the mixture hindered stringent spectral assignments (data not shown).

## Conclusion and Future Perspectives

The aim of this study was to examine if ionic liquids supplements, specifically choline-based ones, can support discovery of bioactive secondary metabolites in three distinct fungi – *N. crassa, A. nidulans*, and *A. fumigatus*. The usage of ionic liquid-based supplements has been shown before to greatly impact fungal metabolism, leading to upregulation of the expression of genes coding in secondary metabolism, including some backbone genes, and altering the ensuing extracellular metabolic footprint. Building on this past evidence, choline-based ionic liquids were used as growth media supplements (at concentrations below their MIC, Table 1), testing different anions and concentrations as well. In either fungus, the media supplementation altered the diversity of compounds accumulating extracellularly (Figure 1). The peptidome composition of the obtained crude extracts (inferred by the abundance/diversity of amino acids in the corresponding hydrolyzates) was also impacted by the supplementation (Supplementary Table 1). Specifically, ACC and Aib levels showed increasing trend in *N. crassa* and *A. fumigatus*, respectively (Figure 2). Moreover, these metabolite extracts reduced the metabolic activity of bacterial cells, in some cases leading to cell lysis (Figures 3, 4). Based on the estimated IC_50_ values (Table 2), the supplementation compared to control conditions, increased greatly the bactericidal activity of the derived *N. crassa* extracts, but not those of *A. fumigatus*. At this stage, the observed activity cannot be linked to a specific compound. To pinpoint potential candidates, untargeted MS analyses using the GNPS platform were applied. A total of 52 and 18 compounds were identified in *N. crassa* and *A. fumigatus* extracts derived from the ionic-liquid supplemented cultures, respectively (Figure 5 and Supplementary Table 4). By eliminating compounds of low signal intensity, the most promising candidates potentially produced by *A. fumigatus* are macrolides and terpenes, whereas for *N. crassa* are cyclic peptides, including five depsipeptides; structurally of high pharmacological interest (Table 3). Fractionation of the later, added another cyclic peptide to the pool of compounds annotated through the GNPS tool; likely of low abundance in the crude extract. Analysis of their whole chemical landscape highlighted, however, a weak sample deconvolution with many compounds present in the three fractions. Through their direct query in the NRPro database, five additional hits of cyclic peptides (including one depsipeptide) were found (Supplementary Table 4).

The usage of GNPS as a dereplication strategy clearly showed that a rich diversity of structures can be generated under an ionic liquid stimulus. It allowed for a rapid comparison of the collected MS data, to obtain a “holistic” view of the chemical space of the fungal extracts, getting one step closer to the identification of novel bioactive metabolites. Its effectiveness can be illustrated by two related examples: diversity of secondary metabolites in *Botryosphaeria mamani* upon medium supplementation with histone deacetylase inhibitors (Triastuti et al., 2019), and in *Penicillium nordicum*, which completed with isotope labeling analyses, led to identification of 69 unknown metabolites (Hautbergue et al., 2019). The tool is subjected to the availability of similar structures in the GNPS databases (as highlighted by additional identifications in the fractions when using NRPro); all the identifications proposed herein remain putative and further confirmation is therefore required. Database search tools, e.g., Mascot, usually used for the MS/MS identification of linear peptides are not directly applicable to cyclopeptides or depsipeptides that generate very complex fragmentation patterns. In addition, >300 NPAAs can be incorporated into fungal NRPs, further enlarging the associated chemical space. None of the compounds putatively identified (Supplementary Tables 4, 5) contains either ACC or Aib, irrespectively of their detection in the hydrolyzates of the crude extracts/fractions. This is likely due to the lack of similar structures in the GNPS and NRPro databases. Besides, it reveals that the chemical space of either extract remains to be fully disclosed. Despite these limitations, specifically the GNPS tool exposed the most promising candidates – cyclic (depsi)peptides of *N. crassa*, setting foundations for their isolation and identification in the near future.

The data attained highlight the capacity of *N. crassa* to generate a rich portfolio of cyclic peptide-based metabolites, with high pharmacological interest. In the genome of *N. crassa*, only four putative NRPS genes have been assigned, none, however, has been linked to the produced metabolite to date. Preliminary tests suggest that three of these genes suffered upregulation in the supplemented medium compared to control (data not shown). Due to the scarcity of NRPS genes in *N. crassa* genome, ionic-liquid supplementation shows matchless potential to link each NRPS to its peptide-product(s), deserving focused analysis soon.

## Data Availability Statement

The datasets presented in this study can be found in online repositories. The names of the repository/repositories and accession number(s) can be found below: EBI – MTBLS5072.

## Author Contributions

CSP and GG supervised the project. CSP supervised the interpretation of data and prepared the final version of the manuscript. All authors have made substantial contributions to the acquisition, analysis and interpretation of data, and contributed to the drafting of the manuscript.

## Conflict of Interest

The authors declare that the research was conducted in the absence of any commercial or financial relationships that could be construed as a potential conflict of interest.

## Publisher’s Note

All claims expressed in this article are solely those of the authors and do not necessarily represent those of their affiliated organizations, or those of the publisher, the editors and the reviewers. Any product that may be evaluated in this article, or claim that may be made by its manufacturer, is not guaranteed or endorsed by the publisher.

## Footnotes

1
http://proteowizard.sourceforge.net

2
http://gnps.ucsd.edu

3
https://web.expasy.org/nrpro/
