# Supplementary material for: Untargeted Metabolomics Sheds Light on the Secondary Metabolism of Fungi Triggered by Choline-Based Ionic Liquids
Source: Front Microbiol. 2022 Jul 25;13:946286. doi: 10.3389/fmicb.2022.946286 (PMC9361774; doi:10.3389/fmicb.2022.946286)
Supplement: Supplementary file 1 [file Table_1.DOCX]

**Fig S1**. Chromatographic analyses of hydrolysates of the fractions obtained from the N. crassa crude extract derived from cultures grown in media containing the Choline chloride supplement, and collected at retention times of A) 15.6, B) 17.3, C) 19.6, D) 29.7 and E) 33.6 minutes.

| 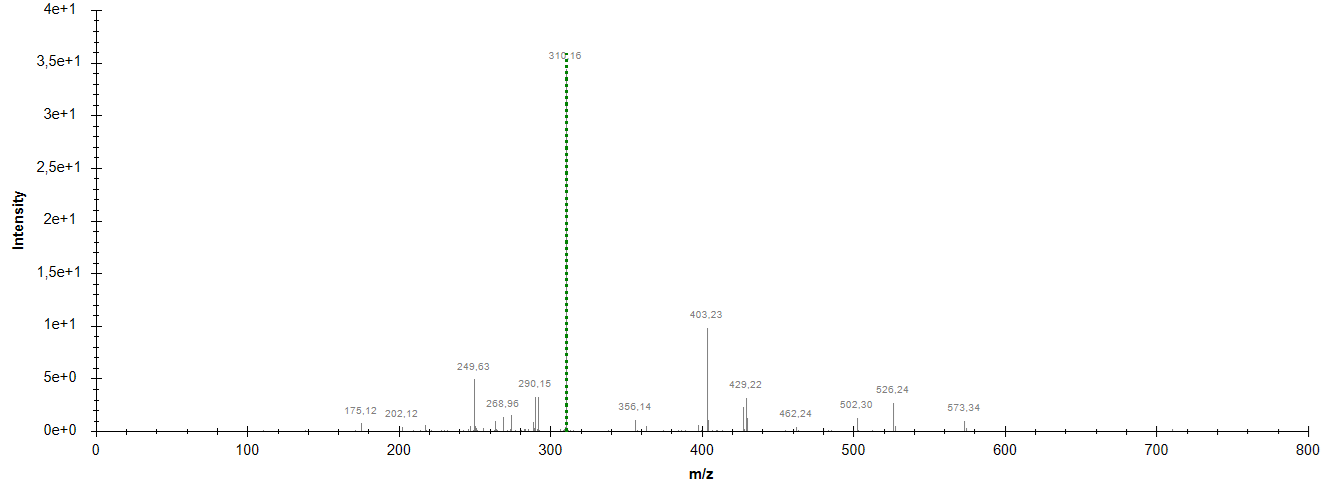 |  |
| --- | --- |
| m/z 310.165 from G1 matching Guangomide A | m/z 323.184 from G1 matching Cyclo(Leu-Ser-Glu-Thr-Thr-D-Leu) |
|  |  |
| m/z 455.731 from G1 matching Arbumelin | m/z 413.241 from G2 matching Cyclotheonamide E3 |
|  |  |
| m/z 445.247 from G2 matching Nostophycin |  |
| **Fig S2.** Spectra matching the putative identification matches found in the NRPro. | |

**Table S1.** Amino acid content analysis of A. nidulans, A. fumigatus and N. crassa crude extracts derived from cultures grown in media with or without (i.e. negative) supplementation, choline chloride (ChoCl) or choline decanoate (ChoDec) at 50% or 80% of the MIC. Values depict the percentage (%) of each amino acid relative to the total amino acid amount, presented as Mean±SD.

|  | ***N. crassa*** | | ***A. fumigatus*** | | | ***A. nidulans*** | | |
| --- | --- | --- | --- | --- | --- | --- | --- | --- |
|  | **Negative** | **ChoCl** | **Negative** | **ChoDec 50%** | **ChoDec 80%** | **Negative** | **ChoCl 80%** | **ChoDec 80%** |
| **ACC** | 1.3±0.6 | 7.0±1.1 | 0.5±0.2 | 0.3±0.3 | 0.2±0.2 | 0.1±0.1 | 0.2±0.1 | 0.9±0.3 |
| **Aib** | 0.3±0.4 | 0.1±0.0 | 3.8±4.8 | 3.9±4.2 | 11.4±15.1 | 0.1±0.1 | 0.2±0.1 | 1.4±0.5 |
| **Alanine** | 20.2±5.8 | 28.7±1.7 | 24.7±12.1 | 17.5±9.5 | 16.5±10.8 | 31.8±1.6 | 29.3±4.4 | 8.5±3.9 |
| **Glycine** | 3.7±2.5 | 0.9±0.2 | 9.4±7.8 | 1.9±1.3 | 4.2±5.0 | 21.7±8.0 | 31.8±6.6 | 20.0±4.5 |
| **Isoleucine** | 6.1±2.1 | 3.8±3.2 | 0.5±0.9 | ND | ND | 0.5±0.7 | ND | 1.6±0.3 |
| **Leucine** | 19.0±5.6 | 4.8±2.8 | 1.2±2.4 | 0.1±0.2 | 0.2±0.3 | 15.7±1.9 | 13.1±2.8 | 4.9±1.5 |
| **Methionine** | ND | ND | 2.3±2.6 | 0.8±1.0 | 1.2±2.0 | ND | 0.3±0.7 | ND |
| **Phenylalanine** | 0.1±0.2 | 0.5±0.0 | 1.0±0.9 | 3.2±1.8 | 2.2±1.3 | 1.4±1.1 | 0.5±0.3 | 4.2±1.9 |
| **Proline** | ND | 0.1±0.2 | 6.6±8.8 | 4.4±5.6 | 7.3±9.4 | 0.0±0.1 | 0.3±0.2 | 0.7±0.7 |
| **Valine** | 38.8±10.7 | 51.0±3.9 | 4.6±2.8 | 2.5±2.6 | 4.0±3.1 | 18.7±2.9 | 18.9±1.0 | 12.9±5.2 |
| **Cystine** | 1.5±2.1 | ND | 0.3±0.6 | 0.2±0.4 | ND | ND | 0.1±0.1 | ND |
| **Serine** | 1.9±1.5 | 0.6±0.2 | 3.4±5.6 | 1.3±1.7 | 1.0±1.7 | 1.2±0.6 | 0.3±0.3 | 6.2±2.4 |
| **Threonine** | ND | 0.1±0.1 | 0.9±1.8 | 0.1±0.2 | 0.1±0.3 | ND | ND | 0.8±0.6 |
| **Tyrosine** | 3.8±0.2 | 1.5±0.1 | 27.2±9.9 | 24.1±9.0 | 21.3±12.2 | 6.7±3.7 | 3.6±2.6 | 24.9±12.7 |
| **Aspartic acid** | 2.0±1.0 | 0.6±0.2 | 10.1±5.0 | 39.7±12.5 | 30.0±5.9 | 1.8±0.7 | 0.9±0.1 | 9.3±0.7 |
| **Glutamic acid** | 1.0±1.2 | 0.4±0.2 | 2.3±4.6 | 0.0±0.1 | 0.3±0.6 | 0.3±0.4 | 0.2±0.2 | 3.4±0.6 |
| **Arginine** | 0.3±0.4 | 0.1±0.1 | 0.7±1.5 | ND | 0.0±0.1 | ND | 0.2±0.5 | 0.4±0.2 |
| **Histidine** | ND | ND | 0.1±0.3 | ND | ND | ND | ND | ND |
| **Lysine** | ND | ND | 0.4±0.9 | ND | ND | ND | ND | ND |
| **% peak area assigned to standards** | | | | | | | | |
| **% matched** | 35.9±7.4 | 54.0±5.7 | 67.6±9.0 | 70.6±5.4 | 69.1±6.1 | 93.1±2.8 | 95.7±1.3 | 73.0±4.3 |
| **% not matched** | 64.1±7.4 | 46.0±5.7 | 32.4±9.0 | 29.4±5.4 | 30.9±6.1 | 6.9±2.8 | 4.3±1.3 | 27.0±4.3 |

*^a^*ND – not detected; ACC - 1-aminocyclopropane-1-carboxylic acid; Aib - α-aminoisobutyric acid

**Table S2.** Comparison of significant differences of MTT measurements from bacteria grown in the presence of different concentrations of the crude extracts from *N. crassa* and *A. fumigatus* cultures grown in media with or without (*i.e.* negative) supplementation. The one-way ANOVA test was performed to each condition relative to control (bacteria grown with no extract) and in the between different conditions at same concentration.

| **Fungal**  **Strain** | **Bacterial**  **Strain** | **Comparison (condition vs control)** | **p-value (One-way ANOVA test)** | **Comparison (same concentration different conditions)** | **p-value (one-way ANOVA test)** |
| --- | --- | --- | --- | --- | --- |
| **N. crassa** | ***E. coli*** | **ChoCl 80%**  [1 mg∙mL^-1^]****  [0.5 mg∙mL^-1^]****  [0.25 mg∙mL^-1^]** | 6,86E^-8^  2,69E^-6^  0,0021 | **Negative control**  **vs ChoCl 80%**  [1 mg∙mL^-1^]****  [0.5 mg∙mL^-1^]****  [0.25 mg∙mL^-1^]** | 2,82E^-11^  2,50E^-5^  0,0037 |
|  | ***S. aureus*** | **Negative control**  [1 mg/mL]** | 0,00342 | **Negative control**  **vs ChoCl 80%**  [1 mg∙mL^-1^]****  [0.5 mg∙mL^-1^]****  [0.25 mg∙mL^-1^]**  [0.125 mg∙mL^-1^]* | 6,45E^-9^  9,30E^-5^  2,77E^-9^  0,024 |
|  |  | **ChoCl 80%**  [1 mg∙mL^-1^]****  [0.5 mg∙mL^-1^]****  [0.25 mg∙mL^-1^]****  [0.125 mg∙mL^-1^]** | 6,01E^-6^  6,04E^-6^  1,67E^-5^  0,0026 |  |  |
| **A. fumigatus** | ***E. coli*** | **Negative Control**  [1 mg∙mL^-1^]****  [0.5 mg∙mL^-1^]****  [0.25 mg∙mL^-1^]***  [0.125 mg∙mL^-1^]** | 1,02E^-6^  1,59E^-5^  1,78E^-4^  0,0025 | **Negative control**  **vs ChoDec 50%**  [1 mg∙mL^-1^]****** | 0,0095 |
|  |  | **ChoDec 50%**  [1 mg∙mL^-1^]****  [0.5 mg∙mL^-1^]***  [0.25 mg∙mL^-1^L]** | 8,09E^-5^  4,37E^-4^  0,0067 | **Negative control**  **vs ChoDec 80%**  [1 mg∙mL^-1^]****** | 0,0026 |
|  |  | **ChoDec 80%**  [1 mg∙mL^-1^]****  [0.5 mg∙mL^-1^]***  [0.25 mg∙mL^-1^]* | 7,29E^-5^  5,41E^-4^  0,041 |  |  |
|  | ***S. aureus*** | **Negative control**  [1 mg∙mL^-1^]***  [0.5 mg∙mL^-1^]**  [0.25 mg∙mL^-1^]* | 6,05E^-4^  0,0019  0,023 | n**s** | ns |
|  |  | **ChoDec 50%**  [1 mg∙mL^-1^]***  [0.5 mg∙mL^-1^]**  [0.25 mg∙mL^-1^]* | 2,81E^-4^  0,0013  0,019 |  |  |
|  |  | **ChoDec 80%**  [1 mg∙mL^-1^]**  [0.5 mg∙mL^-1^]* | 0,0039  0,019 |  |  |
|  |  |  |  |  |  |

* *p* ≤ 0.05 ** *p* ≤ 0.01 *** *p* ≤ 0.001 **** *p* ≤ 0.0001; ns *p* > 0.05. *^a^ ns, non-significant*

**Table S3.** Accession hyperlinks for *N. crassa* and *A. fumigatus* jobs in GNPS platform, specifically Molecular networking and *in silico* DEREPLICATOR+ tool.

| **Fungal strain** | **Link** |
| --- | --- |
| *A. fumigatus* | Molecular network: https://gnps.ucsd.edu/ProteoSAFe/status.jsp?task=3ad96b8f862649f7b093b108c1b830d6 |
|  | DEREPLICATOR+: https://gnps.ucsd.edu/ProteoSAFe/status.jsp?task=e4fc67fa7f934ef59f641332e29d51b0 |
| *N. crassa* | Molecular network: https://gnps.ucsd.edu/ProteoSAFe/status.jsp?task=90a9e46b3366476da0c0be310bec167a |
|  | DEREPLICATOR+: https://gnps.ucsd.edu/ProteoSAFe/status.jsp?task=f2c4cc99f6514113afccf8655c3b34e0 |

| **Table S4.** Putative identifications retrieved by using molecular networking analysis and compound dereplication in GNPS, from spectral match (*) and *in silico* tool DEREPLICATOR+. For *N. crassa*, isolated fractions (G1, G2 and G3) as well as crude (G4) extracts, whereas for *A. fumigatus*, extracts from cultures supplemented with Choline Decanoate at 50% (G1) and 80% (G2) of the MIC were analyzed. | | | | | | | |
| --- | --- | --- | --- | --- | --- | --- | --- |
| **Putative identification** | **RT (sec)** | **Exact mass** | **m/z found** | **Adduct** | **Class** | **Reported**  **activity** | **Reference** |
| ***Aspergillus fumigatus*** | | | | | | | |
| Antibiotic A 59770A | 2334 | 1000.63 | 501.321 | M+2H | Macrolide | Pesticidal agents | (Hoehn et al., 1990) |
| Dolabelide C | 4340.22 | 796.497 | 797.508 | M+H | Macrolide | Antitumor | (Suenaga et al., 1997) |
| Roflamycoin | 3104.03 | 738.455 | 370.236 | M+2H | Macrolide | Antifungal; antiprotozoaric | (Schlegel and Thrum, 1971; Han et al., 2021) |
| Brasilionolide B | 4159.99 | 1180.7 | 591.356 | M+2H | Macrolide | Antifungal | (Chiu et al., 2016) |
| Efomycin G | 1878.16 | 1010.58 | 506.297 | M+2H | Macrolide | Antibacterial; antitumor | (Wu et al., 2013; Supong et al., 2016; Gui et al., 2019) |
| Karlotoxin 3 | 3807.66 | 1324.85 | 663.43 | M+2H | Polyketide | Hemolytic | (Van Wagoner et al., 2010) |
| Aeruclynamide D | 2809.06 | 603.06 | 604.774 | M+OH | Cyclohexapeptide | Antiparasite | (Portmann et al., 2008) |
| Dihydroxylycopene diglucoside diester | 1101.17 | 790.538 | 396.275 | M+2H | Carotenoids | Not reported | (Takaichi et al., 2001) |
| Leukotriene E4 methyl ester* | 3872.23 | 459.22 | 459.313 | M+H | Eicosanoid | [Immunomodulation](https://www.sciencedirect.com/science/article/pii/S095232780700141X) | (Cohen et al., 2002) |
| Soyasapogenol A | 1657.02 | 475.378 | 474.339 | M+H | Triterpenoid | Antitumor | (Kitagawa et al., 1985; Zhang and Popovich, 2008) |
| Xanthomonic acid | 1266.77 | 468.31 | 467.312 | M+H | Terpenoid | Antitumor | (Saleh et al., 2016) |
| 7α,27-Dihydroxycholesterol* | 787.783 | 401.342 | 399.367 | M+H-H2O | Steroid | Not reported | (Brown and Jessup, 1999) |
| 9-(Z)-Octadecenamide | 3893.79 | 563.55 | 563.325 | 2M+H | Fatty acid | Hypolipidemic; antibacterial; antifungal | (Cheng et al., 2010; dos Reis et al., 2019) |
| 13-(Z)-Docosenamide | 1371.23 | 338.34 | 339.24 | M+H | Fatty acid | Antifungal; antibacterial | (dos Reis et al., 2019) |
| Auriculoside B | 2807 | 1214.64 | 608.328 | M+2H | Pregnane glycoside | Antitumor | (Zhang et al.) |
| CID 102041441 | 3361.29 | 810.477 | 406.244 | M+2H | Pregnane glycoside | Not reported | (Deng et al., 2010) |
| Otophylloside B | 4153.65 | 922.529 | 462.27 | M+2H | Pregnane glycoside | Antiepiletic | (Yang et al., 2017) |
| Fosinopril | 2757.34 | 564.308 | 564.331 | M+H | Synthetic compound | Angiotensin-converting enzyme inhibitor | (Sica et al., 1998) |
| ***Neurospora crassa*** | | | | | | | |
| Amphoteronolide B | 894.328 | 778.414 | 390.212 | M+2H | Macrolide | Not reported | (MASAMUNE, 1988) |
| Marinisporolide B | 3025.43 | 692.414 | 347.216 | M+2H | Macrolide | Not reported | (Kwon et al., 2009) |
| Aldgamycin K | 1667.11 | 696.367 | 349.19 | M+2H | Macrolide | Antibacterial (*S.aureus*) | (Wang et al., 2016) |
| Levorin A0 | 1371.81 | 1110.59 | 556.301 | M+2H | Macrolide | Not reported | (Szwarc et al., 2015) |
| Levorin A3 | 3210.69 | 1092.58 | 547.296 | M+2H | Macrolide | Antifungal | (Pawlak et al., 2005; Szczeblewski et al., 2017) |
| Antibiotic A 59770A | 2334 | 1000.63 | 501.321 | M+2H | Macrolide | Pesticidal agents | (Hoehn et al., 1990) |
| Dideoxy-Sandramycin | 1806.18 | 1188.56 | 595.289 | M+2H | Cyclic depsipeptide | Antitumor | (Boger and Chen, 1997) |
| Myxochromide S2 | 2679.72 | 736.416 | 369.216 | M+2H | Cyclic depsipeptide | Not reported | (Wenzel et al., 2005) |
| Discokiolide A | 1682.41 | 1026.51 | 514.263 | M+2H | Cyclic depsipeptide | Antitumor | (Tada et al., 1992) |
| Chaiyaphumine D | 3385.91 | 644.296 | 645.3 | M+H | Cyclic depsipeptide | Not reported | (Grundmann et al., 2014) |
| Chlorodestruxin | 1524.11 | 629.319 | 315.666 | M+2H | Cyclic depsipeptide | Anti-insecticidal | (Gupta et al., 1989) |
| SF-1902-A4 | 4248.2 | 667.452 | 334.735 | M+2H | Cyclic lipodepsipeptide | Antibacterial | (Omoto et al., 1981) |
| SCH-378199 | 3062.28 | 562.819 | 562.885 | M+2H | Depsipeptide | Not reported | (Hegde et al., 2001) |
| Syringostatin A | 1259.88 | 1178.59 | 590.3 | M+2H | Cyclic lipodepsipeptide | Antifungal | (Sorensen et al., 1996) |
| Val-Val-Pro-Val-Pro-Asn* | 1797.39 | 651.396 | 326.705 | M+2H | Peptide | Not reported | In-house library from GNPS |
| Actinomycin C2 | 2184.16 | 1284.64 | 643.329 | M+2H | Peptide | Not reported | (Nam et al., 1998) |
| Annosquamosin A | 803.485 | 848.41 | 425.212 | M+2H | Peptide | Not reported | (Li et al., 2010) |
| Pepsin S 735A | 4211.02 | 685.463 | 343.737 | M+2H | Peptide | Protease inhibitor | (Morishima et al., 1970; OMURA et al., 1986) |
| Actinomycin F4 | 3819.11 | 1256.64 | 629.327 | M+2H | Peptide | Not reported | (Kanchanasin et al., 2020) |
| Keramamide A | 966.764 | 942.441 | 472.228 | M+2H | Peptide | Not reported | (Kobayashi et al., 1991) |
| Desferrioxamine X5 | 920.406 | 598.369 | 300.193 | M+2H | Peptide | Not reported | (Konetschny-Rapp et al., 1992) |
| Halo-toxin | 2558.88 | 626.343 | 314.178 | M+2H | Peptide | Not reported | (Kajimoto et al., 1989) |
| API II/AgrD2 | 2175.6 | 744.348 | 373.181 | M+2H | Peptide | Autoinducing peptide | (M. et al., 2010) |
| Antamanide* | 3397 | 573.85 | 573.5 | M+2H | Peptide | Antidote | (Wieland et al., 1968; Azzolin et al., 2011) |
| Fru-Leu-Ile* | 1087.43 | 407.239 | 407.192 | M+H | Peptide | Not reported | In-house library from GNPS |
| Ile-Pro-Ile* | 1554.93 | 342.239 | 342.266 | M+H | Peptide | Not reported | In-house library from GNPS |
| Mollamide B | 1667.11 | 696.367 | 349.19 | M+2H | Cyclic peptide | Antimalarial, antivirus, antitumor | (Donia et al., 2008) |
| Wewakazole | 3484.76 | 1140.54 | 571.278 | M+2H | Cyclic peptide | Antitumor | (Nogle et al., 2003; Gogineni and Hamann, 2018) |
| Cycloreticulin B | 3439.96 | 834.395 | 418.204 | M+2H | Cyclic peptide | Not reported- | (Wélé et al., 2008) |
| Pseudostellarin C | 725.799 | 812.443 | 407.23 | M+2H | Cyclic peptide | Tyrosinase inhibitor; antitumor | (Morita et al., 1994) |
| L-Tryptophan | 1091.51 | 204.09 | 409.17 | 2M+H | Amino acid | Building block | In-house library from GNPS |
| Pregn-5-ene-3,8,11,12,14,20-hexol, 9Cl, 11-Ac,3-O-dig-dig-cym-ole | 3117.61 | 1000.56 | 501.28 | M+2H | Terpene glycoside | Not found | Not found |
| 3,12,14,17-Tetrahydroxypregn-5-en-20-one, 12-Ac,3-O-ole-can-ole | 2908.46 | 824.456 | 413.233 | M+2H | Terpene glycoside | Not found | Not found |
| 3,16-Dihydroxycucurbita-5,20(22)-diene-11,24-dione, 3-O-xyl-rham | 3337.38 | 748.44 | 375.225 | M+2H | Terpene glycoside | Not found | Not found |
| 3,11,14-Trihydroxycard-20(22)-enolide, 3-O-dig-dig | 3034.65 | 678.398 | 340.207 | M+2H | Terpene glycoside | Not found | Not found |
| Periplocoside M | 2798.55 | 604.361 | 303.189 | M+2H | Terpene glycoside | Not reported | (ITOKAWA et al., 1988) |
| Stauntoside K | 3029.26 | 762.419 | 382.215 | M+2H | Terpene glycoside | Not reported | (Yu et al., 2013) |
| Batatoside A | 4929.79 | 1268.66 | 635.335 | M+2H | Terpene glycoside | Not reported | (Yin et al., 2008) |
| Orizabin XIV | 4948.15 | 1120.6 | 561.308 | M+2H | Glycolipid | Antitumor; β-1-3-glucan synthase inhbitor; antibacterial | (Pereda-Miranda and Hernández-Carlos, 2002) |
| Sublanceoside K1 | 4950.56 | 1082.57 | 542.29 | M+2H | Terpene glycoside | Not reported | (Warashina and Noro, 2006) |
| Tuberoside h2 | 2759 | 780.43 | 391.222 | M+2H | Terpene glycoside | Not reported | (Warashina et al., 2011) |
| CID 102041441 | 3361.29 | 810.477 | 406.244 | M+2H | Terpene glycoside | Not reported | (Deng et al., 2010) |
| Hirundigoside D | 3154.49 | 978.525 | 490.272 | M+2H | Terpene glycoside | Anti-inflammatory | (Lai et al., 2016) |
| Lentinan | 876.36 | 1134.37 | 1135.38 | M+H | Glycoside | Not reported | (Zong et al., 2012) |
| Eutypellacytosporin B | 3044.43 | 714.398 | 358.204 | M+2H | Terpene | Not reported | (Zhang et al., 2019) |
| Hoyacarnoside A | 3126.63 | 956.534 | 479.274 | M+2H | Terpene | Not reported | (ABE et al., 1999) |
| Betamethasone* | 3343.06 | 393.208 | 391.212 | M+H | Terpene | Not reported | (Takeoka et al., 2001) |
| Corticosterone* | 4894.41 | 347.222 | 347.215 | M+H | Terpene | Not reported | (Steiger and Reichstein, 1938) |
| Progesterone | 916.55 | 315.232 | 313.852 | M+H | Terpene | Not reported | (Salhanick et al., 1952) |
| Leukotriene E4 methyl ester* | 3872.23 | 459.22 | 459.313 | M+H | Eicosanoid | [Immunomodulation](https://www.sciencedirect.com/science/article/pii/S095232780700141X) | (Cohen et al., 2002) |

| **Table S5.** Putative identifications retrieved by using NRPro for *N. crassa* most intense MS/MS obtained for the fractions (G1, G2 and G3) | | | | | | | | |
| --- | --- | --- | --- | --- | --- | --- | --- | --- |
| **Putative identification** | **RT (sec)** | **Exact mass** | **m/z found** | **Class** | **Reported activity** | **Special**  **amino acid** | ***p*-value** | **Reference** |
| Fraction G1 | | | | | | | | |
| Guangomide A | 1094.38 | 618.326 | 310.165 | Cyclodepsipeptide | Weak antibacterial activity | LNMeAla and D-NMePhe | 6.20E^-5^ | (Sy-Cordero et al., 2011) |
| Cyclo(Leu-Ser-Glu-Thr-Thr-D-Leu) | 1379.11 | 644.338 | 323.184 | cyclic peptide | Not reported | D-leucine | 1.07E^-4^ | Not found |
| Arbumelin | 1775.52 | 909.460 | 455.731 | cyclic peptide | Not reported | D-amino acids | 0.019 | (Mao et al., 2015) |
| Fraction G2 | | | | | | | | |
| Cyclotheonamide E3 | 2894.53 | 857.4874 | 413.241 | cyclic peptide | Serine protease  inhibitor | D-alloisoleucine | 0.004 | (Maryanoff et al., 1993) |
| Nostophycin | 1680.82 | 888.474 | 445.247 | cyclic peptide | Weakly cytotoxic; antimicrobial | novel amino acid Ahoa | 0.008 | (Fujii et al., 1999; Gupta and Vyas, 2021) |
